# Pedro Ramón y Cajal: The Legacy of a Neurohistologist, a Medical Doctor, and a Pathologist

**DOI:** 10.1002/ar.24137

**Published:** 2019-04-29

**Authors:** Santiago Ramón y Cajal, Juan A. de Carlos

**Affiliations:** ^1^ Department of Pathology Vall d'Hebron University Hospital Barcelona Spain; ^2^ Spanish Biomedical Research Network Centre in Oncology (CIBERONC) Barcelona Spain; ^3^ Department of Molecular, Cellular and Developmental Neurobiology Instituto Cajal (CSIC) Madrid Spain

**Keywords:** Ramón y Cajal, neurohistology school, Cajal's disciples

## Abstract

The work of Santiago Ramón y Cajal is difficult to understand without knowing the personalities of Justo, his father, and his brother Pedro. His father practically forced the two brothers to study medicine. Thanks to that, Santiago was able to combine his artistic talent with histology and wonderfully describe cerebral architecture. Pedro was a faithful brother and above all a friend of Santiago, and they worked together for years. Pedro was able to demonstrate the theories of his brother in nonmammalian amniotes, concluding that the basic elements of the nervous system are common to these animals and he provided images that served Santiago to formulate the theory of dynamic polarization. Pedro, who decided to remain in the shadow of his brother, was a very complete doctor, pathologist and gynecologist, who made interesting contributions in all these fields and above all was a great humanist who left an important personal and scientific legacy. Anat Rec, 2019. © 2019 The Authors. *The Anatomical Record* published by Wiley Periodicals, Inc. on behalf of American Association of Anatomists. Anat Rec, 303:1189–1202, 2020. © 2019 The Authors. *The Anatomical Record* published by Wiley Periodicals, Inc. on behalf of American Association of Anatomists.

## JUSTO RAMÓN: THE FATHER AND THE “MAKER” OF THE RAMÓN Y CAJALS

**Figure 1:**
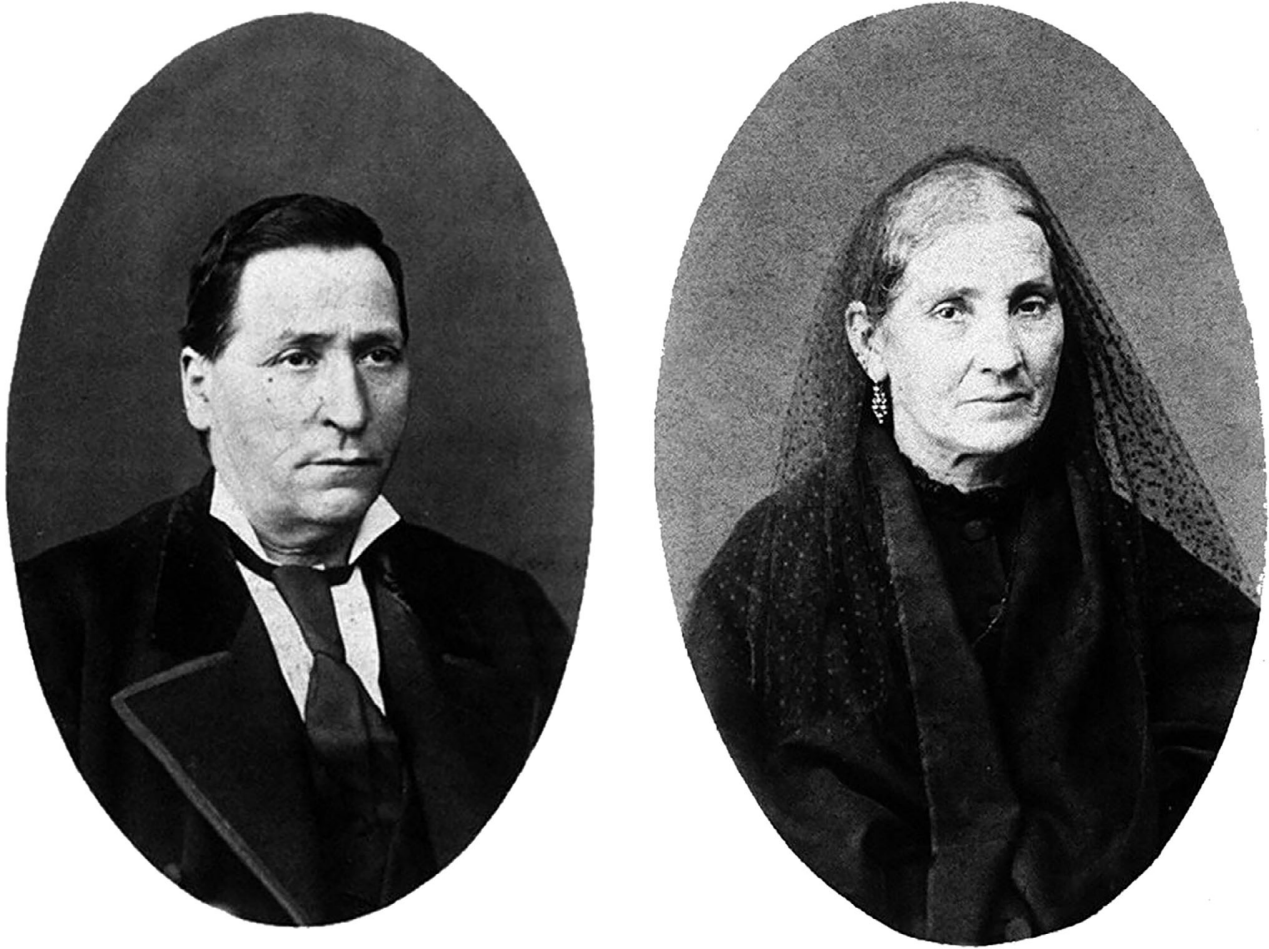
Portraits of Justo Ramón and Antonia Cajal.

Life was not easy for the young Justo Ramón Casasús, who was the third son of a humble family of farmers (his father Antonio Ramón; his mother Rosa Casasús; and three brothers Antonio, Simón, and Mariano) from Larrés, a town close to Jaca, in the province of Huesca, in the north of Spain (Garcés Romeo, 1987; De Carlos, 2001). Justo, who had been born in 1822, had to help his parents with farm work, working the land and shepherding from a very young age. However, the boy had very high aspirations and, at just 16 years old, he left the family home and moved to a nearby town, Javierrelatre, where he began working as a surgeon's apprentice. This was his first contact with medicine and he stayed with the discipline for the rest of his life. During his first years at this work, Justo taught himself to read using books from his employer's library. At the age of 21, with some savings, he set out on foot to Zaragoza, where he found a job in a barbershop in the Arrabal neighborhood. He combined work with study and managed to pass his high‐school degree with good marks. Still working in the barbershop, he passed the entrance exams to a place as a physician's assistant in the *Hospital Provincial*: this was a permanent position. Not content with this new success, Justo decided to continue studying to become a surgeon. However, in 1845, he had to leave because a new law abolished the teaching of medicine in Zaragoza, which stopped his studies. Justo refused to give up and decided to take a risk. He left his job in the hospital and moved to Barcelona to continue his study, where he worked once again in a barbershop in the Sarriá neighborhood, which allowed him to pay his way during that time. Once he finished his studies, he returned to Larrés and began courting Antonia Cajal, whom he would marry in September 1849 (Fig. 1). The newlyweds settled in the town of Petilla de Aragón, where Justo obtained a position as a second‐class surgeon. It was in this town in Navarra, on 1 May 1852, that they had their first child, whom they named Santiago. In October 1853, Justo returned to Larrés, where he worked for two years. On 23 October 1854, Pedro, their second son, was born. Justo moved house several more times, until he found a permanent home in the city of Zaragoza. From Larrés he moved to the town of Luna, in the province of Zaragoza, then to Valpalmas where his daughters Pabla and Jorga were born. Justo left his wife and children in Valpalmas and moved to Madrid; there, he studied for a medical degree, which he obtained in 1858 at the age of 35. He returned in 1860, having achieved his goal, and he applied for the position of community doctor in the town of Ayerbe, in the province of Huesca: he got the position and moved there. Finally, Justo moved to Zaragoza in 1870 after passing a national exam to become a provincial welfare doctor. Shortly afterward, don Genaro Casas, dean of the faculty of medicine, awarded him the position of acting professor of dissection. With this post, he was able to directly help his two sons study anatomy (Alonso and De Carlos, 2018).

Justo Ramón Casasús, endowed with an indomitable will, was a self‐made man. Once he set his goals, he devoted himself entirely to them, be it clinical medicine in his private clinic, providing welfare medical services, or in his teaching role in the faculty of medicine. He had a stern, authoritative character, and tried to instill in his sons a love of study; moreover, impassioned with his profession, he wanted them to become doctors at all costs. We all know that they achieved this, albeit not without a great effort and some headaches: Santiago was not a good student and had a great vocation for painting and drawing, and Pedro ran away from home in what cannot be classed as a mere adventure, as his absence lasted seven years. We can certainly say that don Justo played a key part in his sons' education and that if they made something of their lives, they owed it to their father (De Carlos, 2001; Alonso and De Carlos, 2018).

## PEDRO AS A CHILD AND TEENAGER

Pedro Ramón y Cajal had a quiet childhood and a lifelong close relationship with his brother and sisters. He was a good student until his last year of high school, and unlike his brother Santiago, there was no major mischief until he was a teenager. Of note, and clearly reflecting his strong, persevering character, was his little‐known “escapade” at the end of the school term that caused more than a mild headache for his father don Justo. Pedro did indeed finish his high school studies at the *Instituto de Huesca* aged 17. However, he failed one subject in his final year and did not dare to face his father with this failure. So he made a pact with a friend and the two of them ran away from home. This escape cannot really be considered harmless mischief, as he left Spain and made it all the way to Bordeaux, where he boarded a sailboat named “Queen,” headed for South America. This getaway lasted seven years, which he spent in Uruguay and Argentina, with many ups and downs. The first problems began on the boat: Pedro and his friend, finding themselves without any money, had boarded as stowaways. Discovered on board during the crossing, they were locked up and keelhauled as a punishment. After passing this “test,” they were allowed to continue the journey. The crossing lasted three months, much longer than expected, so food and water was scarce; this made the men very irritable and they argued easily. Pedro got into a fight with an Italian whom he wounded with a knife, and the man's friends wanted to throw Pedro overboard, but one of the sailors intervened and managed to save his life. However, he did not escape punishment and spent several days in the stocks.

**Figure 2:**
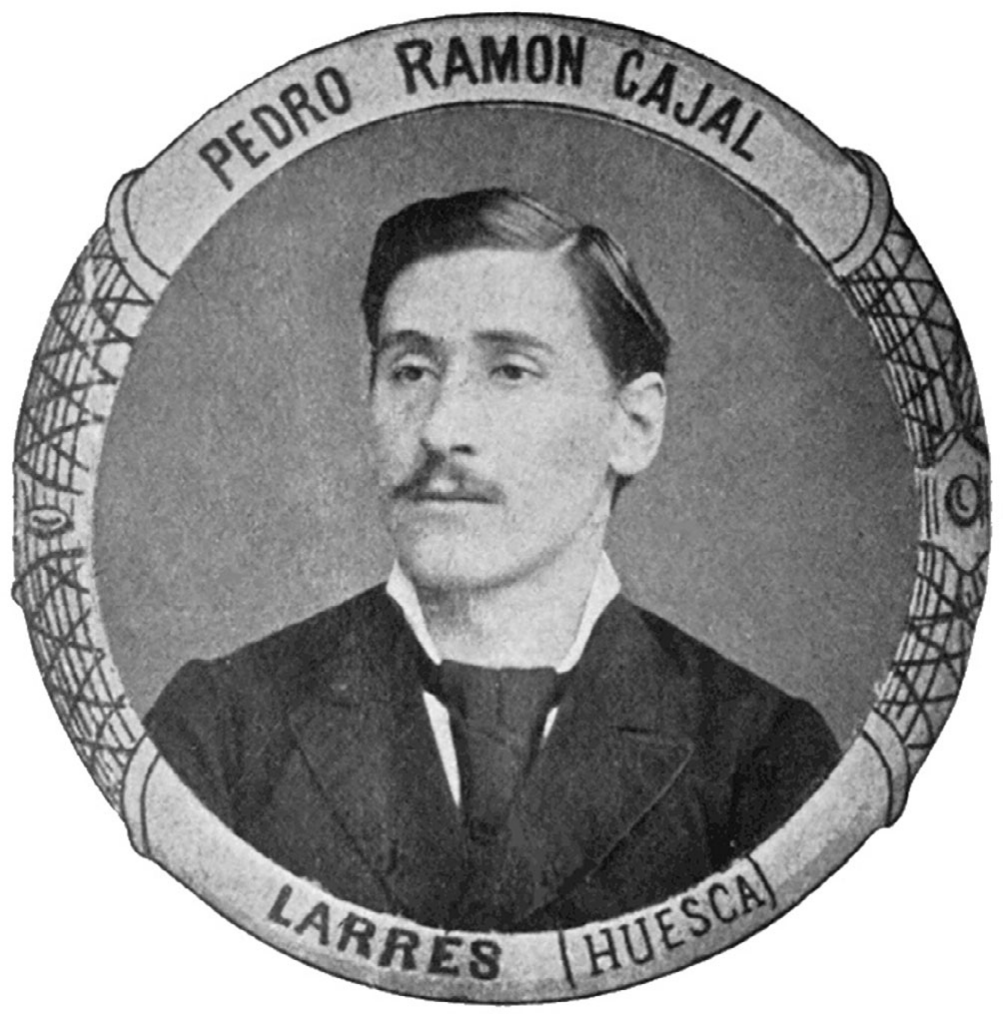
Pedro Ramón y Cajal: University graduation photograph.

In a political environment (see Box 1) of war between neighboring countries and practically of civil war, Pedro Ramón y Cajal arrived in Uruguay and enrolled in the revolutionary ranks. He served as a soldier in civil conflicts and traversed the eastern prairies in several campaigns; in one encounter, he was wounded. However, he seemed to love the Pampas and one day he found himself working as secretary to a Uruguayan leader, Timoteo Aparicio. Pedro reached such a position as he was able to read and write, skills that the native people had not mastered. After seven years of adventures, Pedro and an Italian friend of his agreed to leave Uruguay and decided that it would be a good idea to take Coronel Aparicio's horse and gun with them. They were caught and tried for this felony and sentenced to death. All the while, don Justo was unaware that his son Pedro was in such serious trouble. Fortunately, this was not the case for the family of his Italian friend, who, hearing of their son's predicament, were able to intervene on his behalf through the Italian consulate, shortly before he was due to be executed. The Italian consulate alerted the Spanish consulate that there was also a Spanish citizen awaiting execution: they intervened and managed to save him from certain death. Thanks to this consular intervention, he was able to leave the country and return to Spain (De Carlos, 2001). Once in Spain, and under great pressure from Justo, his father, Pedro began his medical studies (Fig. 2). According to his grandchildren, he never talked about his South American adventure and had a particularly strong Catholic faith and love for Our Lady of the Pillar (image of the Virgin Mary kept in the cathedral of Zaragoza and venerated in Aragón). In fact, this devotion to Our Lady of the Pillar is said to have arisen from the day he was returning to Zaragoza by train: at one of the stops, he got off to have lunch and escaped an explosion on the train. They say that Pedro was astonished by how lucky he had been and he attributed his luck to the beloved Virgin Mary. On a personal level, he was described as humble, simple, and compassionate. He was very close to his patients and many anecdotes have been told by patients and families of him paying for their medical treatment and helping them in their domestic situation.

**BOX 1. Sociopolitical situation in Uruguay and Paraguay, 1870–1880.**

In the midst of the War of the Triple Alliance against Paraguay (1865–1870), Uruguay, to the east, was living moments of great political unrest, with terrible epidemics such as cholera and extreme poverty. In February 1868, the revolution by the Blanco Party (the White Party or National Party) led to the death of two ex‐presidents of the republic, General Flores and don Bernardo Berro, prestigious figures from their respective parties. That same year, General Lorenzo Batlle was elected president of Uruguay. He was from Montevideo and the son of a wealthy Catalan businessman. Batlle belonged to the Colorado Party and had been minister for war during Flores' government. He was extremely worried about the economic crisis, and in 1869, several Uruguayan banks were closed. Payments were defaulted, there were public demonstrations, and all the while cholera was raging. As if that were not enough, departmental revolutions were taking place. Although some were quickly stamped out, the revolution started in 1870 by Coronel Timoteo Aparicio, a white revolutionary, would last close to two years. The first battles went well for him, with the revolutionaries winning in the Paso Severino and Arroyo Corralito areas, then moving toward Montevideo and seizing control of the Fortaleza del Cerro. But at this point, Aparicio decided to move to the interior of the country and fight General Suárez, who had restructured the government army with contingent troops, especially cavalry. Two large battles took place, with the legal government winning both. In the second battle, the revolutionary army's second in command, General Anacleto Medina, died, aged 80. The revolution led by Timoteo Aparicio ended with the signing of “the peace of April 1872,” a treaty that allowed a period of peace between the two sides.

**Figure 3:**
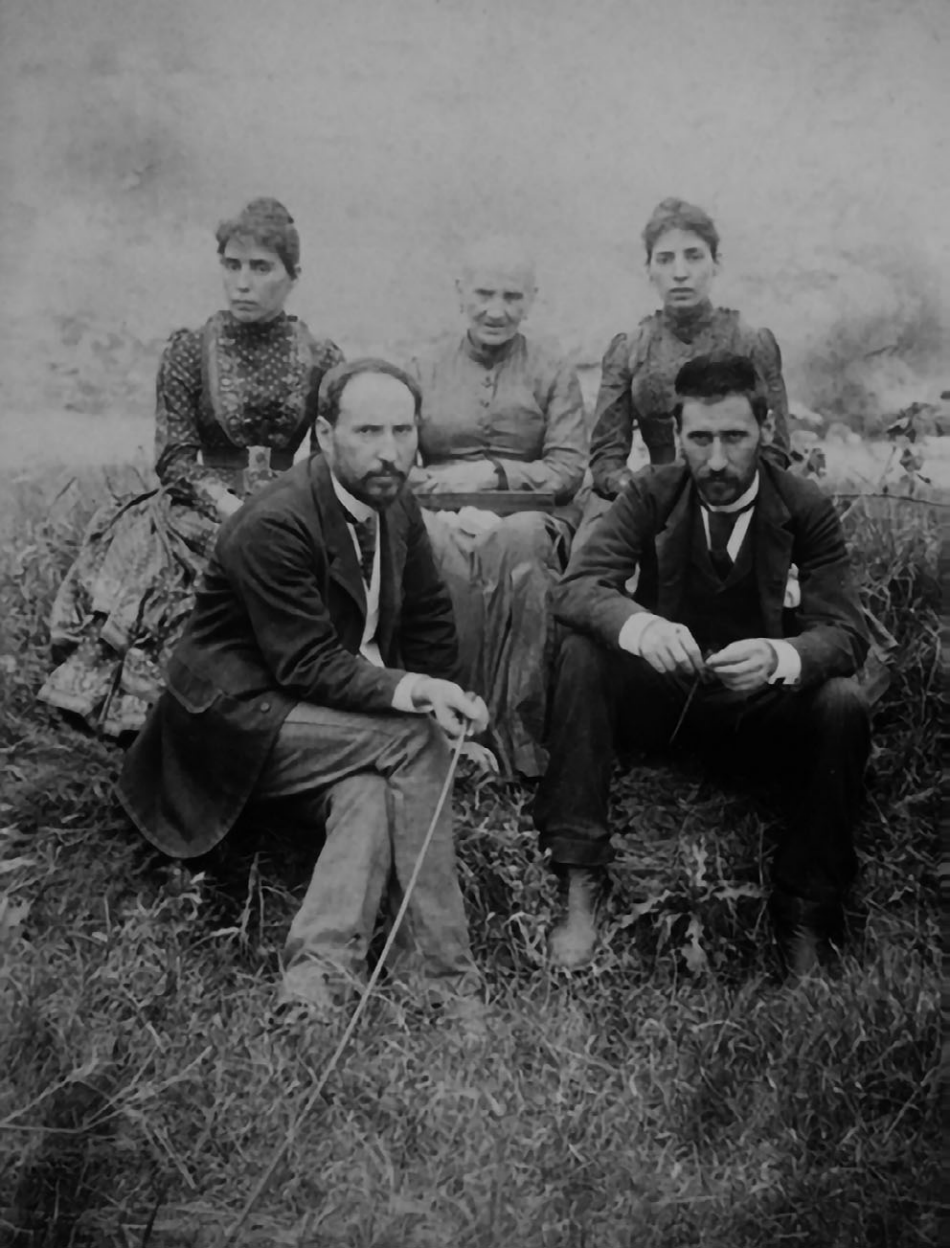
Antonia Cajal surrounded by her four children: Jorja, Pabla, Santiago, and Pedro (*ca*. 1890).

**Figure 4:**
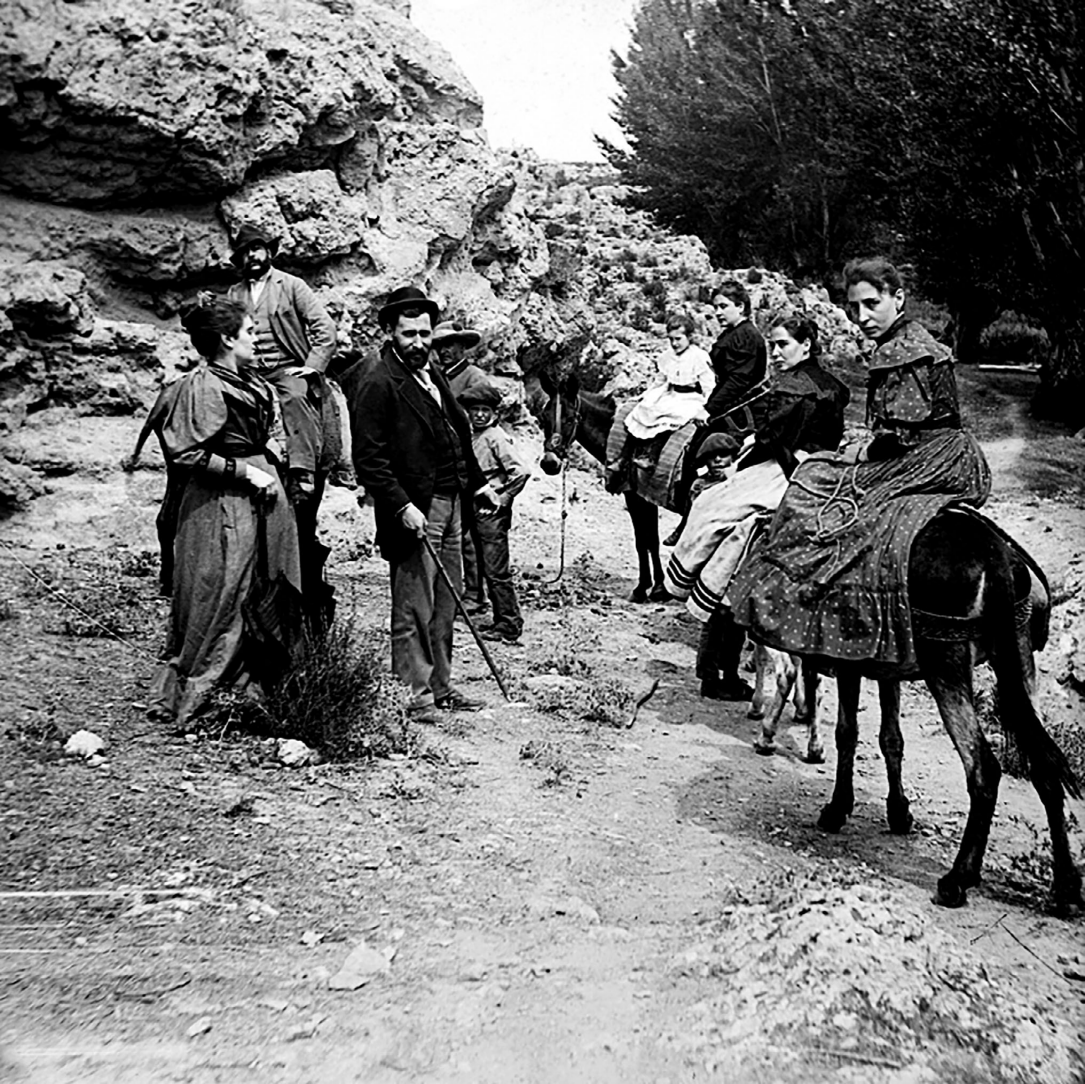
Pedro, his sisters, and the Santiago's wife Silveria. Picture taken by the own Santiago Ramon y Cajal.

He maintained a close relationship with his brothers and his mother (Fig. 3). His brother Santiago used to spend vacations and other periods in Zaragoza with his family. Pedro was also Santiago's personal advisor on medical matters and the complicity between both brothers was enormous throughout his life (Fig. 4).

## STUDYING MEDICINE AND STARTING WORK

Pedro returned to Spain in 1878 aged 24 and began studying medicine at the University of Zaragoza. In 1879, following a competitive exam, he was appointed student intern in anatomy, for which he received a stipend. His degree distinction was dated October 1881 (Fig. 4). Pedro spent the next seven years practicing rural medicine in the towns of La Almolda (1881–1885) and Fuendejalón (1885–1888) in Zaragoza. After that he returned to Zaragoza city, and on 11 February 1888, he married María Vinós Redondo. His first histology works date from this same year, as, drawn to this field, he took advantage of his free time to study the nervous system of any small animal he came across. Over time, he specialized in nonmammalian vertebrates (birds, fish, amphibians, and reptiles) and, guided by his brother Santiago, aimed to help with the latter's research. During this time, he opened a clinic at his home at Calle Blancos, number 4.

## HIS CONTRIBUTIONS TO NEUROSCIENCE

On 3 February 1890, following a competitive exam, Pedro was appointed director of anatomical studies in the faculty of medicine at the University of Zaragoza, performing this role for 3 years and 10 months. He reached his academic peak on 24 November 1890, with a thesis project titled *Investigación de histología comparada de la visión en diversos vertebrados* (A comparative histology study on vision in different vertebrates). The thesis described the key cell types of the visual centers in different species of vertebrates. In his own words, “The outcome of my observations was to demonstrate that all the optic centers of all vertebrates contain the same structural components and that, therefore, the marvelous instrument of vision always corresponds to the same structural formula” (Rodríguez‐Martín, 1985; Alonso and De Carlos, 2018).

**Figure 5:**
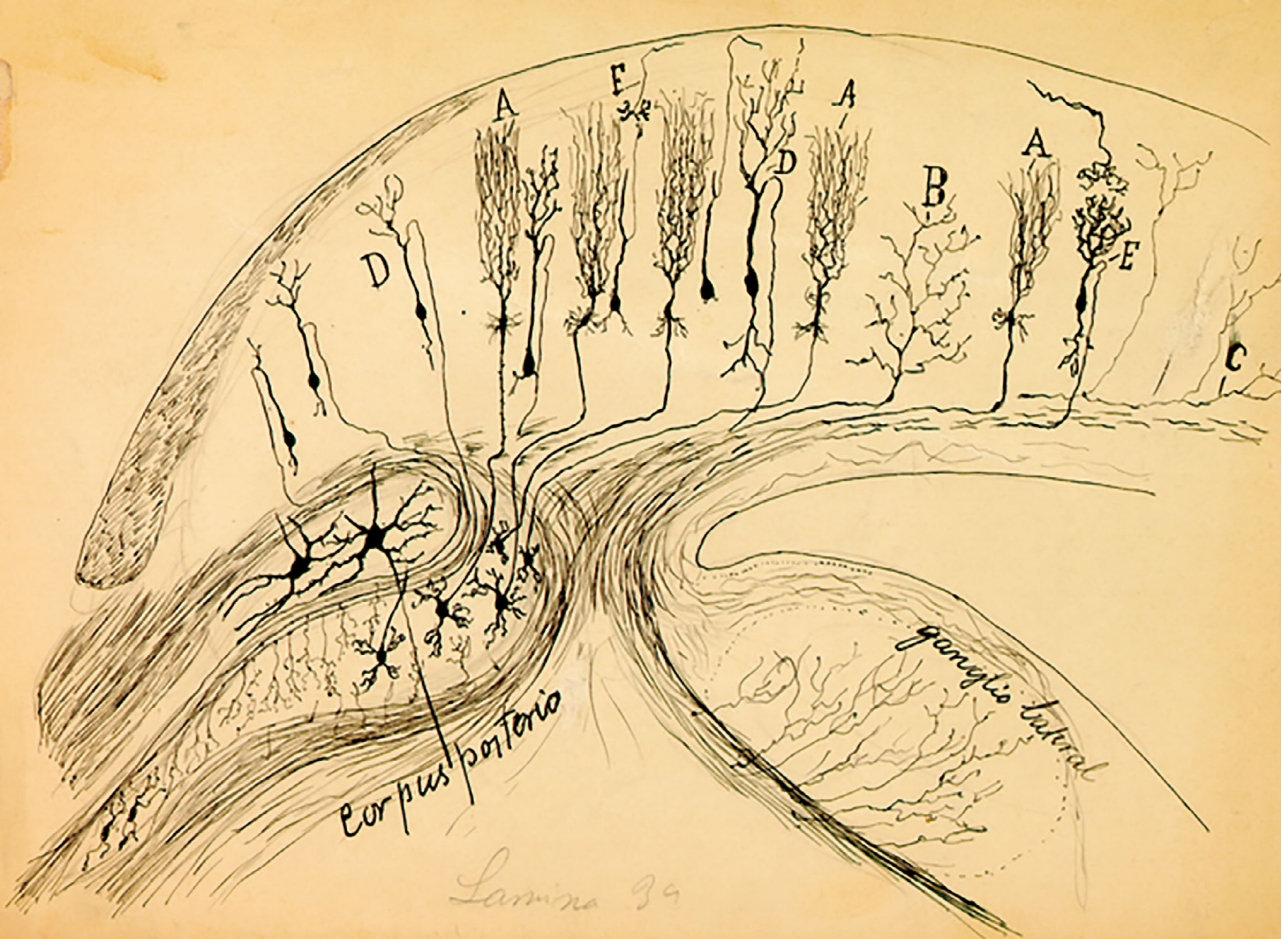
A Pedro's drawing of neurons in the brain of a bird (sparrow). Presumably unpublished as we have not found where it could have been published (about 1898). Represent a caudal part of the optic tectum, jointly with the magnocellular and parvocellular isthmic nuclei (corpus posterior), whose cells interconnect with the characteristic sheperd's crook axon cells of the optic tectum (identified at D). The tectal radially ascending terminal axonal ramifications of the parvocellular isthmic neurons, marked as A, had been described before by his brother Santiago (1891), but their origin was unknown. The “ganglio lateral” is the auditory torus semicircularis. This isthmotectal connectivity remained practically unknown in the field until it was rediscovered 100 years later with modern neuroanatomic methods. Another interesting feature of this drawing is the presence of tectal neurons whose efferent axons take the route of the marginal layer (targeting the diencephalon, as found later).

In 1891, his brother Santiago, working in Barcelona, resumed his work on polarization of the nervous impulse, spurred on by criticisms from Van Gehuchten. Pedro provided him with all his histological preparations on the optic lobe of amphibians, reptiles, and birds. On these magnificent slides, Santiago saw what he had not seen on his own: not a tangle of dendrites, but terminal nerve plexuses (Fig. 5). These observations along with Pedro's findings were crucial in helping Santiago to postulate his Law of Dynamic Polarization.

In January 1895, following an official competition, he was awarded the position of professor of normal and pathological anatomy and histology at the University of Cádiz, where he performed great work, teaching and undertaking research, that continued until 1899 (Ramón y Cajal, 1924; González‐Santander, 1994; González‐Santander, 1998). Several works of histology would come from this period, among them, a monograph on the chameleon brain published in 1896 that, according to him, won him international fame (Ramón y Cajal, 1896):

I do not know why this incomplete study has attracted the attention and admiration of its readers more so than those previously published by me. Before this publication, only Van Gehuchten, professor at Lovaina, was aware of my modest contributions; but after this monograph, Kölliker, Waldeyer, His, and so on, began to take my histological work into consideration. (Ramón y Cajal, 1924)

It seems that Pedro was better known internationally than he had realized. Proof of this was that the eminent German professor Ludwig Edinger, father of comparative neuroanatomy, published in that year, 1896, a discourse on the comparative anatomy of the nervous system (Edinger, 1896), in which he mentioned the works of the two Ramón y Cajal brothers and even reproduced one of Pedro's drawings (De Carlos, 2001).

**Figure 6:**
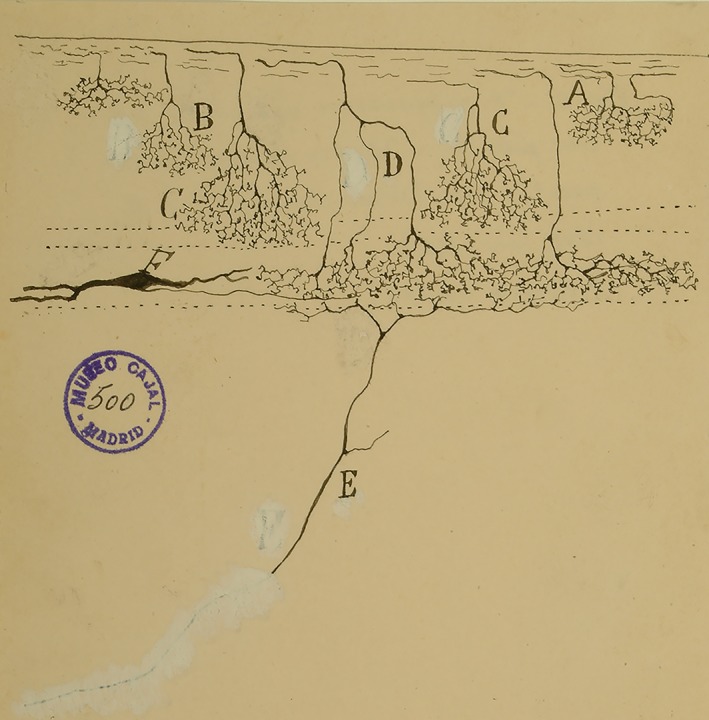
Pedro's drawing (1898) showing tectal dendrites (E and F) and free retinal axon terminals (A–D) supporting the law of dynamic polarization proposed by his brother Santiago.

Let us briefly review his research work to give ourselves an idea of the magnitude and importance of it. Using the Golgi–Cajal method (as he called it, given the modifications introduced by his brother), he studied the brains of nonmammalian tetrapods, describing in particular detail in several papers the cerebral cortex; olfactory bulbs; diencephalic, mesencephalic, and isthmic centers of the lizard (*Lacerta agilis*). He asserted that the forebrain of reptiles was representative of that of mammals, but, being substantially simpler, it provided advantages when it came to studying the fundamental structure of the cerebral cortex. In contrast, the optic lobes were the most complicated organ in the nonmammals. The greater complexity of the cerebrum and cerebellum caused him to conclude that the optic apparatus seems to become simpler in more evolved animals (Rodríguez‐Martín, 1985). Thus, he provided new information on the brain of amphibians and of the chameleon. Of particular importance were his studies of the optic centers of birds and the optic lobes of teleosts. In fact, it was in the optic tectum of birds where he corroborated the terminal plexuses of retinal afferents that spurred his brother to find and consolidate the Law of Neuronal Polarization (Ramón y Cajal, 1898; Ramón y Cajal, 1923) (Fig. 6). When Santiago discovered the reduced silver nitrate staining method, Pedro was the first to use it in nonmammal tetrapods, discovering the masticator motor nucleus of the trigeminal nerve in birds, reptiles, and amphibians. He described the stellate ganglion cells of the deep gray matter of the optic tectum, belonging to the second type of neurons—giant ganglion cells—described by him in all the lower invertebrates. He confirmed, in birds, reptiles, and amphibians, the exact location of the descending column of the trigeminal nerve, which contains a superior portion made of multipolar cells and an inferior portion made of large piriform neurons. Other important works of his include those on the basal ganglion and basal fascicle in amphibians, with its collateral terminal fibers in the brainstem white matter of amphibian larvae. The laws that determine neuronal polarity and connections in the gray matter, postulated by his brother, were variously confirmed by Pedro in the nervous centers of nonmammalian tetrapods and some fishes (Rodríguez‐Martín, 1985).

**Figure 7:**
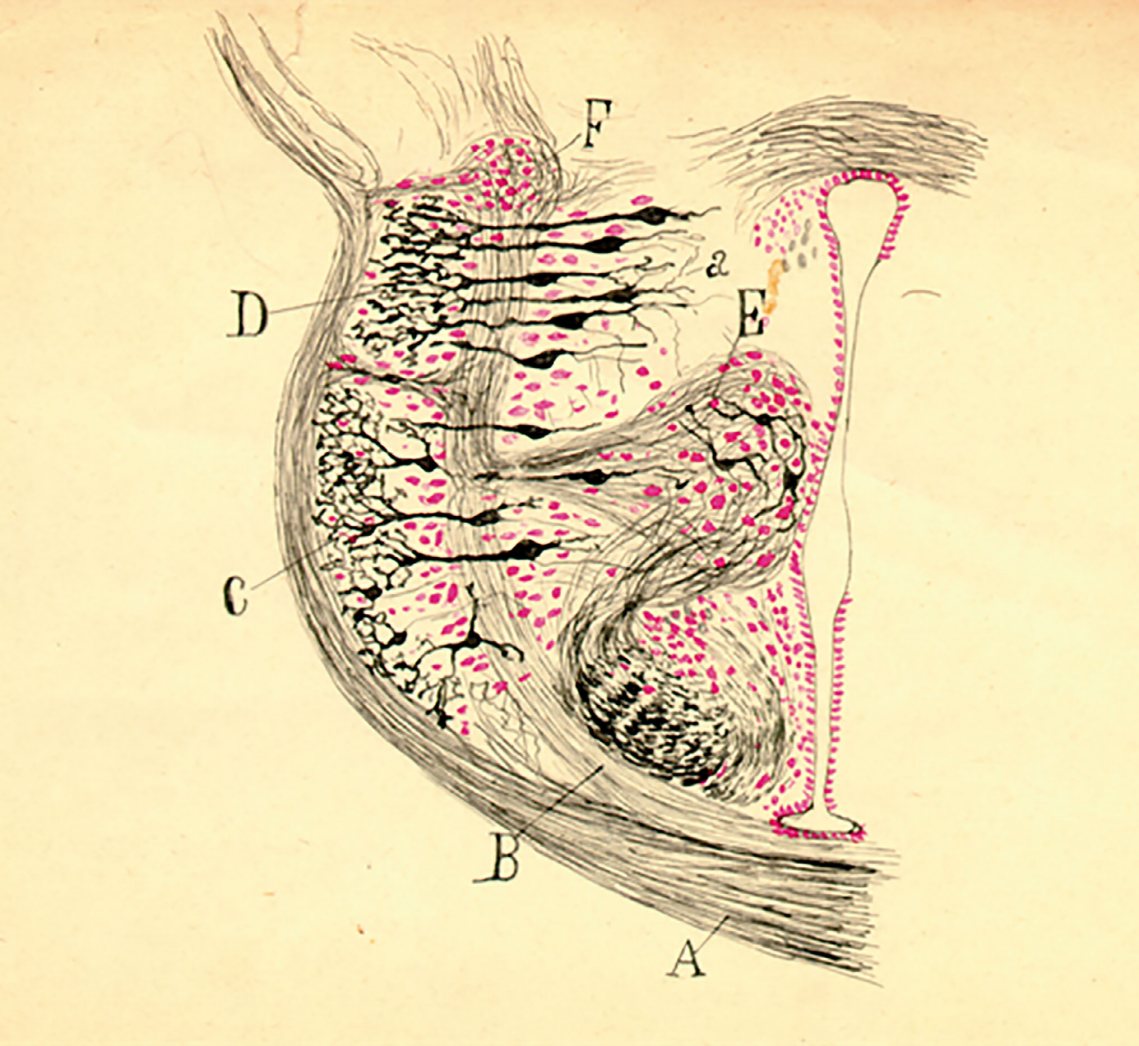
Unpublished colored drawing of a carmin‐counterstained Golgi horizontal section of the reptilian diencephalon, probably lizard (note that the scarce thickness of the periventricular stratum discards the possibility that it belongs to an amphibian).

**Figure 8:**
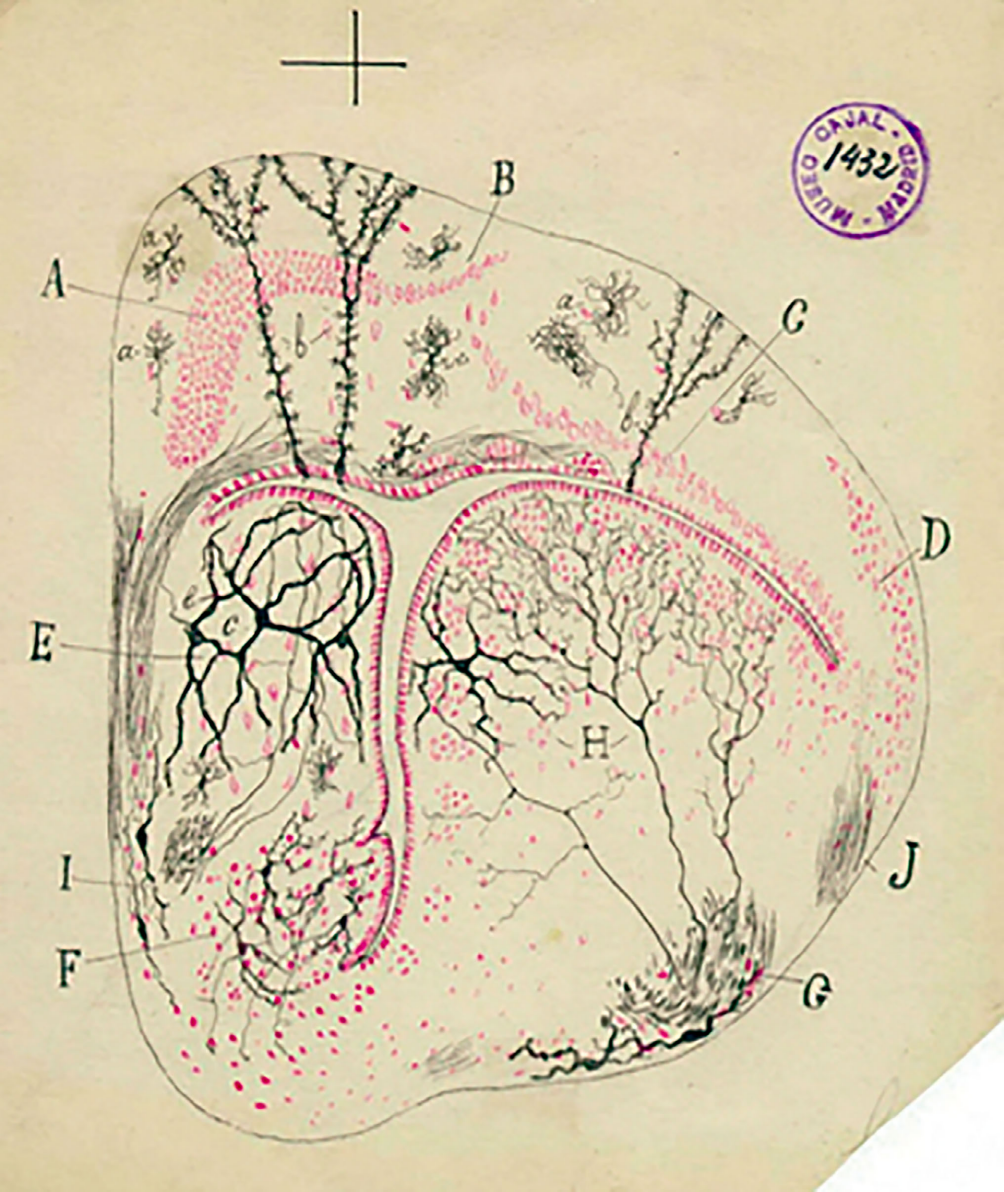
Drawing of a Golgi‐impregnated cross section through the telencephalon in the iguana (1917).

In 1902, he received the Martínez y Molina prize, shared with his brother Santiago, for an extensive histological work *Sobre los centros cerebrales sensoriales en el hombre y los animales* (On the cerebral sensory centers in man and animals) (Alonso and De Carlos, 2018). We must point out that Pedro, although in the background, was always very close to his brother and that the scientific collaboration that he maintained with him consisted, essentially, of confirming all his findings in nonmammals. In his own words, his mission was (Figs. 7 and 8):

…to confirm in the lower vertebrates (amphibians, reptiles and birds) the discoveries in which I participated in an endless correspondence of letters, some of which were like scientific papers. I would send him my findings, and sometimes they would determine his actions or force him to abandon an idea.

In recent times (since the late 1990s), a group of Spanish comparative neuroscientists under the leadership of Luis Puelles (Neuroanatomy, University. of Murcia) and Agustín González (Cell Biology, Complutense University of Madrid) founded a Pedro Ramón Club of Comparative Neurobiology. This is aimed to recognize Pedro's pioneering contributions to this field and maintain his memory fresh by organizing annual neurocomparative symposia (brain morphology, function, development, and evo/devo), organized informally as activities associated in parallel to the meetings of the Spanish Society of Neuroscience. The Club continues its activities at present and has over 90 members, mostly of the younger generation, distributed over various universities or neuroscience research institutes (major nodes in Madrid, Murcia, Alicante, Valencia, Salamanca, Málaga, Lérida, Santiago, Vigo, La Coruña, La Laguna, and Las Palmas de Gran Canaria). Foreign participants are welcome after expression of interest (agustin@bio.ucm.es).

## PEDRO: CLINICIAN AND PATHOLOGIST

When it came to his teachings, others described them as masterful and did not hesitate to call his time at the Cádiz faculty of medicine “the Cajal era”, as found in the album signed by the teachers and students that he was gifted as a tribute, along with a gold plaque, when he left his professorship in 1899. Pedro worked in that position for 4 years and 10 months, until in November 1899 he was awarded, based on a competition of the position of professor of clinical obstetrics and gynecology at the University of Zaragoza (González‐Santander, 1994; González‐Santander, 1998). The price for returning to Zaragoza would be, of course, this change of specialty: Pedro left the teaching of anatomy and histology for the teaching of obstetrics and gynecology, though he continued publishing neurohistological papers on the reptilian, avian, and amphibian brains (1900, 1917, 1918, 1943, and 1946).

Without neglecting his role as professor, Pedro returned to the clinic that he had left when he moved to Cádiz. The clinic was doing well, and he decided to expand it to allow him to attend to all of his patients. He joined together with Dr Ricardo Lozano Monzón, a prestigious thoracic surgeon, and they opened what would be the first private surgical and obstetric clinic in Zaragoza, in *Paseo María Agustín*, opposite *la Puerta de Carmen*. The success of this open‐access clinic at the beginning of the century was such that it soon became too small, prompting the two partners to close it and each open their own clinic. Thus, Dr Lozano opened his clinic in *Paseo Sagasta*, and Pedro, in *Paseo de la Mina* (number 25), in 1925. He completed his new clinic with a general laboratory and a pathology laboratory. In charge of these, he put his son Conrado, who had studied medicine in Madrid, specializing in pathology, with his uncle Santiago.

It should be noted that Pedro was a pioneer in the use of radium to treat cancer in his new clinic in Zaragoza. This radioactive material, bought in 1917, was the first to arrive in Aragon, as at that time neither the faculty of medicine nor the provincial hospital had it. Specifically, the radium was used to treat vulval, cervical, and endometrial cancer. The treatment approach consisted of irradiating the tumor with the aim of halting its growth and reducing its mass, followed by surgical removal and subsequent study of the tumor using the expertise acquired in his histological work. Clearly, the medicine that Pedro practiced would nowadays be done by a whole team of professionals including a family doctor, a gynecologist, a radiologist, and a pathologist.

In October 1924, Pedro retired from his position as university professor, aged 70 (Fig. 8). This was not the end of his practice, though, as he continued working in his private clinic until close to the end of his life, and did not remove himself from the physicians' register until one year before his death (De Carlos, 2001).

What was surprising about Pedro was that despite holding the position of professor in the faculty of medicine to which he had to dedicate hours each day, he had a private clinic with numerous patients, where he saw them and treated their diseases, many of which required surgery. All these jobs would be more than enough to overwhelm any normal professional, yet Pedro still managed to find free time to dedicate to his research. Suffice to add that in 1902 he founded and directed, along with Doctors Lozano and Royo Villanova, the journal *La Clínica Moderna* where, until 1917, he published more than 70 articles on histology and gynecology.

## HIS CONTRIBUTIONS TO PATHOLOGY

His histological work was extensive and did not end with the nervous system, although he continued that work throughout his whole life. He also made interesting contributions on the structure of the uterus, ovarian innervation, the follicular epithelium of the ovary, uterine tuberculosis, ovarian cyst formation, uterine cancer, and others. Without wanting to overload the reader, we would like to briefly mention some of the most salient monographs on reproductive pathology that Pedro wrote during his time as professor in Zaragoza. They included complicated tubal pregnancy, uterine tuberculosis, intrapelvic hematoceles, ovarian papillary cysts, adnexal suppuration, ovarian innervation, anexitis treatments, and the embryonic layer of the uterus, among others (Ramón y Cajal, 1924). Within the study of women's health, he declared, “The study of cancer has been one of my most notable inclinations. On this subject I have published some not unoriginal articles…I dealt extensively with the pathology of this process and the changes induced by radium.”

**Figure 9:**
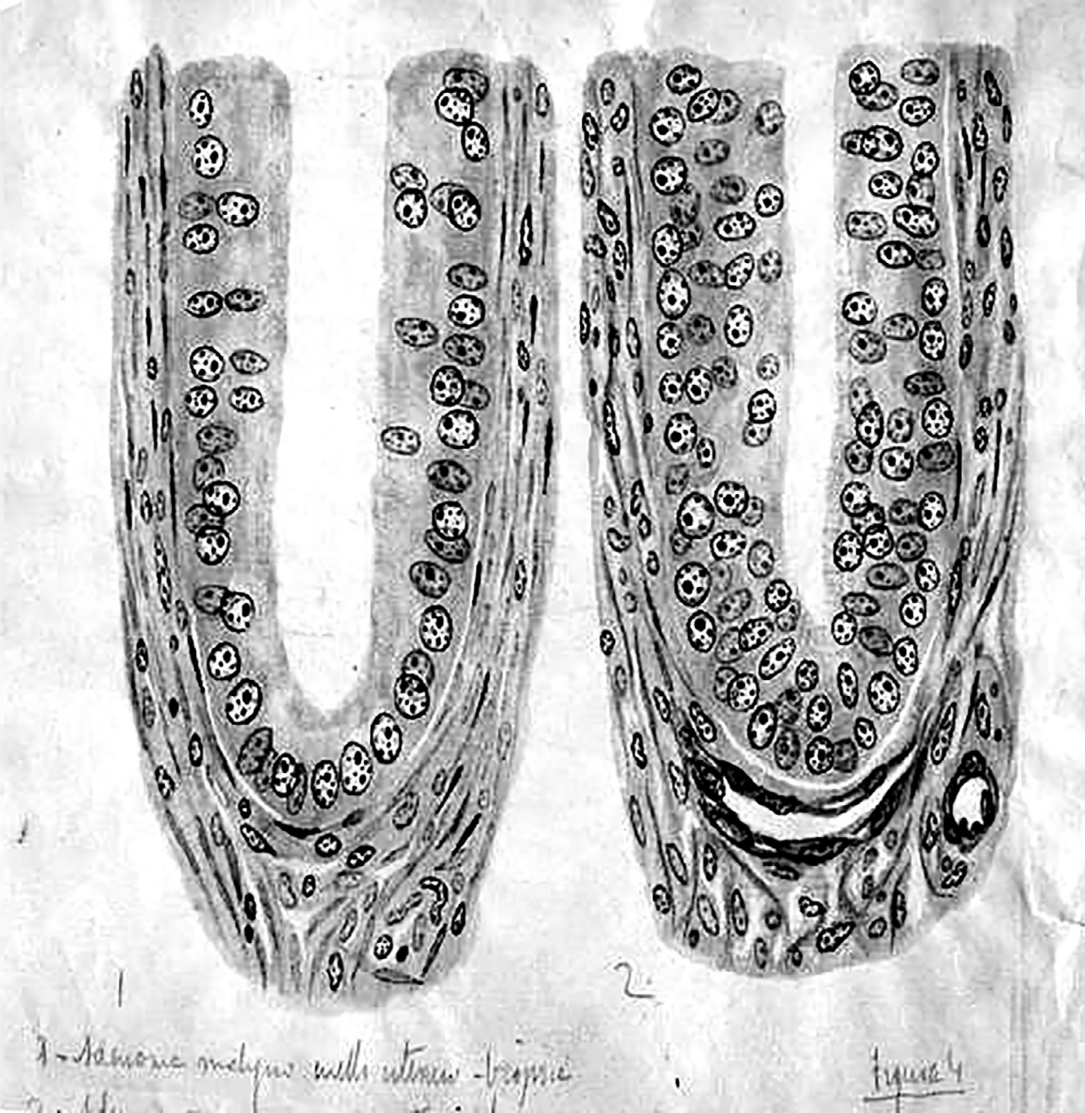
Drawings of a tumor with mildly atypical cells that clinically behaved aggressively. An example of the so‐called nowadays, borderline tumors.

**Figure 10:**
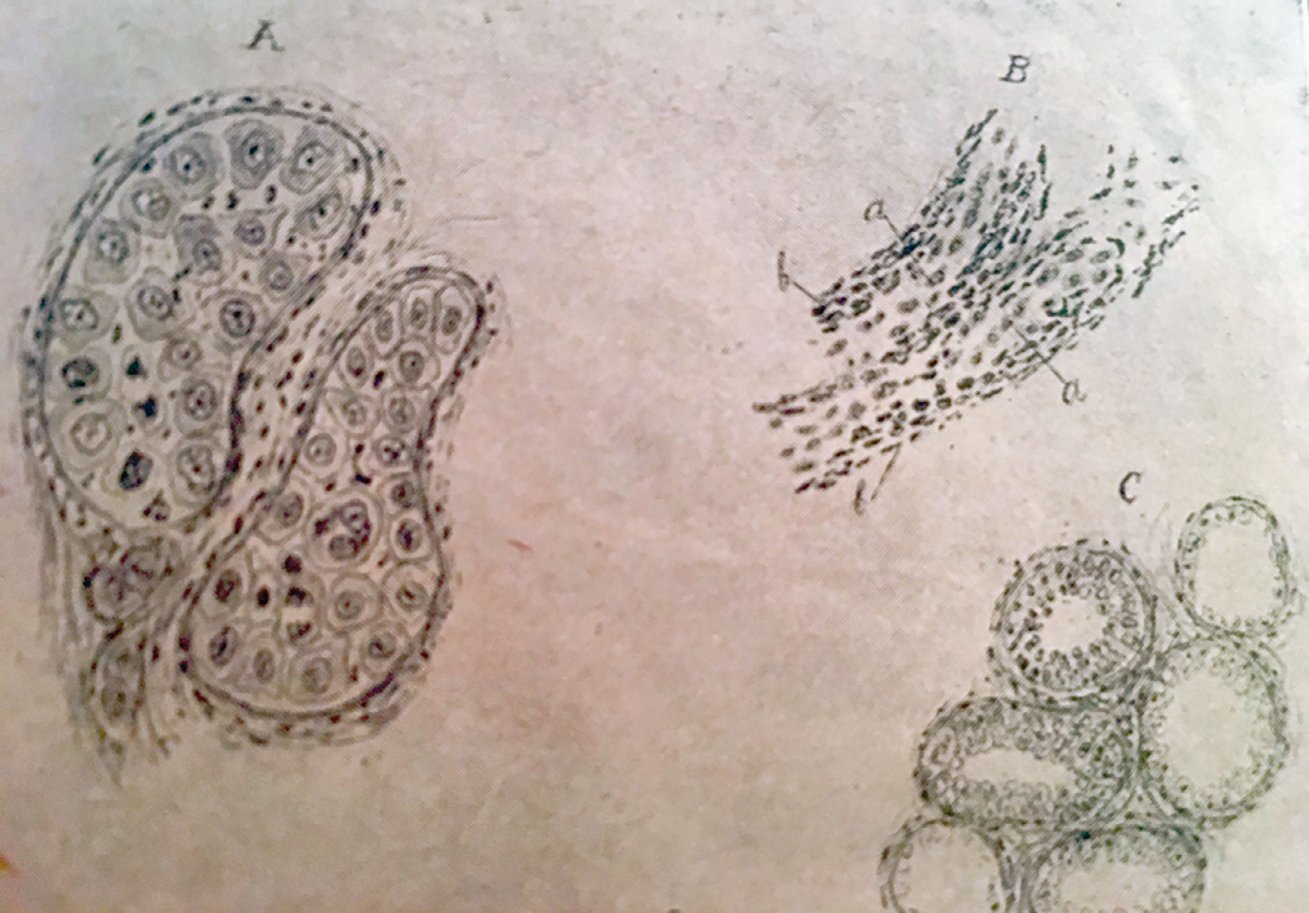
Drawings of a breast tumor that was more poorly differentiated at recurrence.

**Figure 11:**
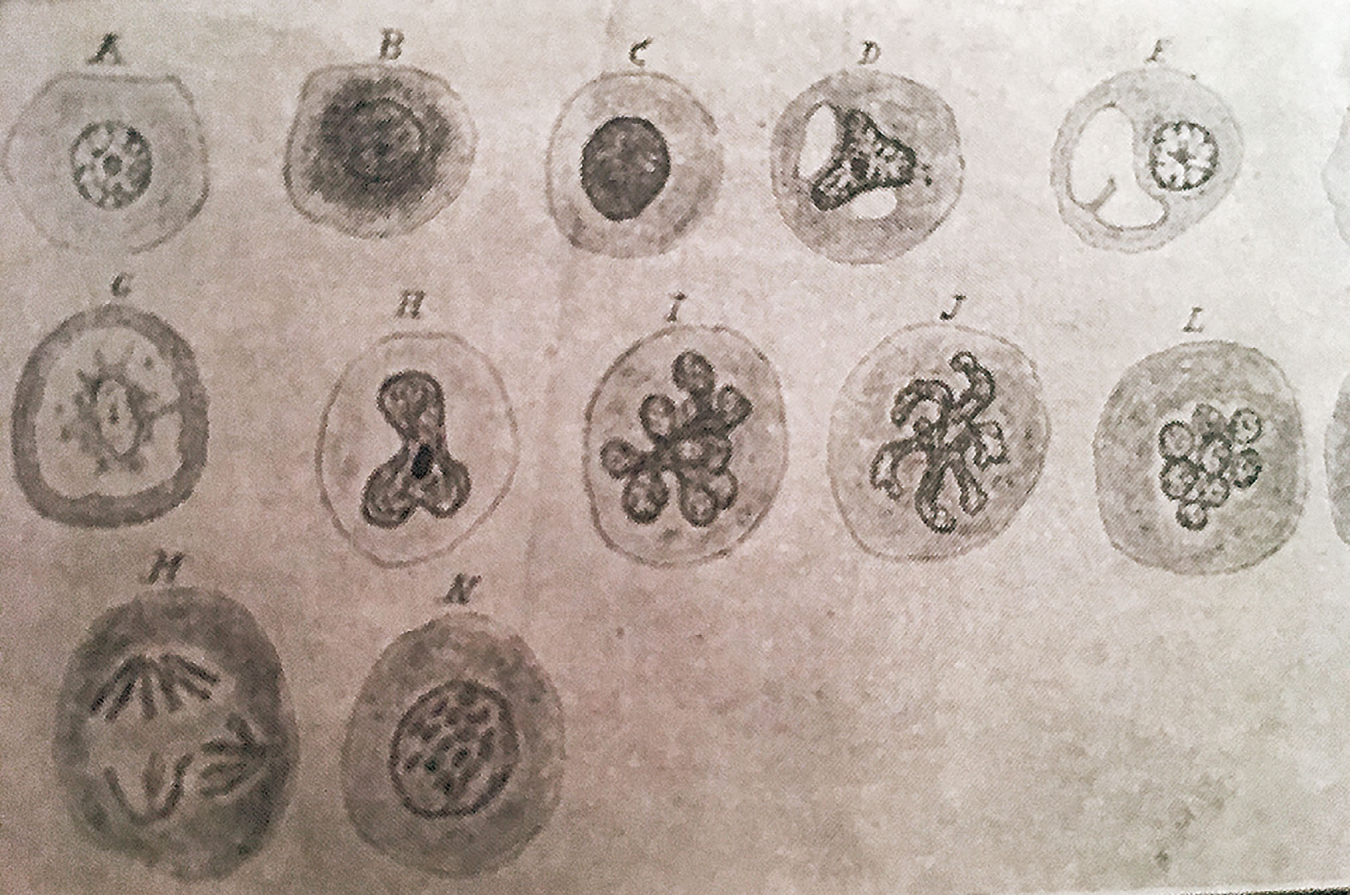
Drawings of the cytological changes induced by radiotherapy.

Of the multiple publications related to pathology, especially gynecological pathology, the following conceptual contributions may be considered some of the most outstanding:

- He was a pioneer in Spain in taking preoperative and intraoperative biopsies as the standard. In fact, some of his publications reveal the importance of such pathological studies under conditions in which rampant chronic inflammatory processes could be confused with malignant tumors.
- Of note among his works on ovarian histology and histopathology were his studies on ovarian innervation, ovarian follicles, and different types of tumor, such as dermoid cysts, in which he discussed their histogenesis.
- He took considerable interest in the apparent contradiction, in some types of tumors, between the severity of the histological changes in the tumor and its clinical behavior. This matter being new and not yet well understood, he published a study on a mucinous ovarian tumor with multiple peritoneal attachments and parietal infiltration, which on cytology showed scanty atypical cells. Thus, he highlighted the clinicopathological discord in some types of tumors that nowadays are misnamed “borderline” (Fig. 9).
- He also pointed out that malignant tumors become progressively more malignant and to illustrate gave cases of recurrent breast cancer with histology that was more atypical and undifferentiated at each recurrence (Fig. 10).
- Finally, we must highlight his contributions, not only clinical but also histopathological, in the study of radium in malignant tumors. He was a pioneer of this technique in Spain and personally treated hundreds of tumors, both benign tumors such as leiomyomas and malignant tumors. With regard to pathology, of note was his histological study on the cytological changes in irradiated cells, from the first instances, through the progression or evolution of the irradiated tissues. As can be seen in Figure 11, he would draw the nuclear and cytoplasmic changes that occurred secondary to radium treatment—nuclei with signs that today we interpret as apoptotic or atypical, which are known to be present in irradiated cells. He also observed and described the highly aggressive and malignant nature of many tumors that do not respond to radiotherapy. According to him, such tumors, refractory to radium, became resistant to radiotherapy in subsequent sessions, and their outcome was worse than those tumors that had not been previously treated.

## HIS CLINICAL CONTRIBUTIONS

**Figure 12:**
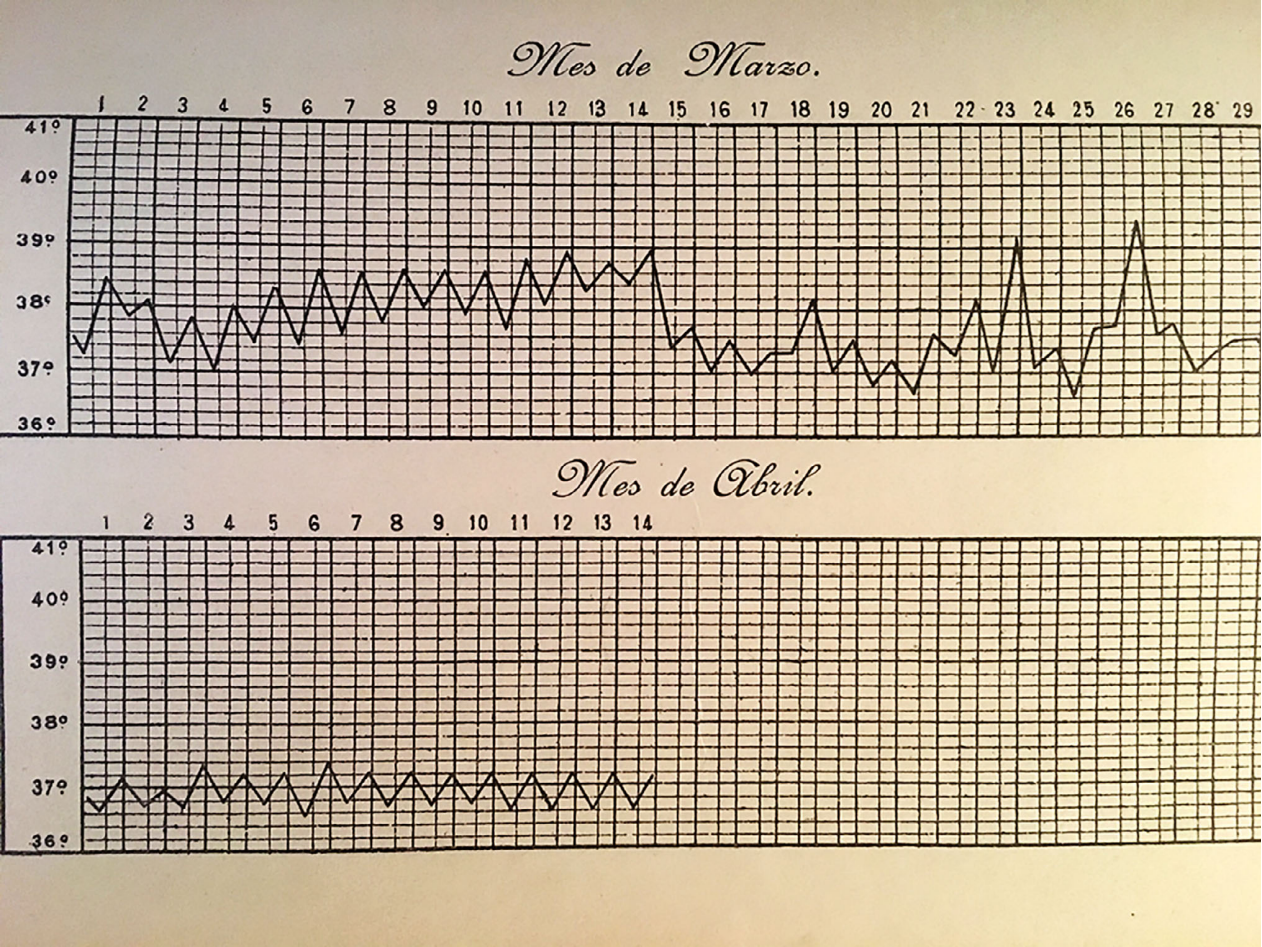
Description of Malta fevers.

But we cannot say that Pedro was outstanding because of his histological and gynecological work alone. He was a man eager to learn and he also loved clinical work, and so he began to observe strange fevers and collections of symptoms that could not be classified to any of the diseases known at the time. Interested by this phenomenon, he studied it and produced as a result his works *Swinging fevers*, *Little‐known febrile processes*, and his magnificent monograph *Mediterranean fevers of Aragon* (Ramón y Cajal, 1904). In 1914, he became a member of the Royal Academy of Medicine of Zaragoza and chose this research topic for his inaugural speech, which he gave under the title *Malta fevers in Aragon* (Fig. 12). It can be said that Pedro Ramón y Cajal, in 1896, was the first clinical researcher in Spain to discover and describe Malta fever, also known as undulating fever, or Brucellosis, which is its modern name. The name Brucellosis came from the British navy doctor Coronel David Bruce who discovered the bacteria that causes the disease; he first observed the signs and symptoms on the Mediterranean Island of Malta. Pedro's disposition for professional practice and interaction with patients led him to study this very common ailment, which was often confused with others such as rheumatism, typhoid fever, febrile anemia, tuberculosis, and malaria. He established the differential diagnosis and workup, allowing it to be treated properly (De Carlos, 2001).

**Figure 13:**
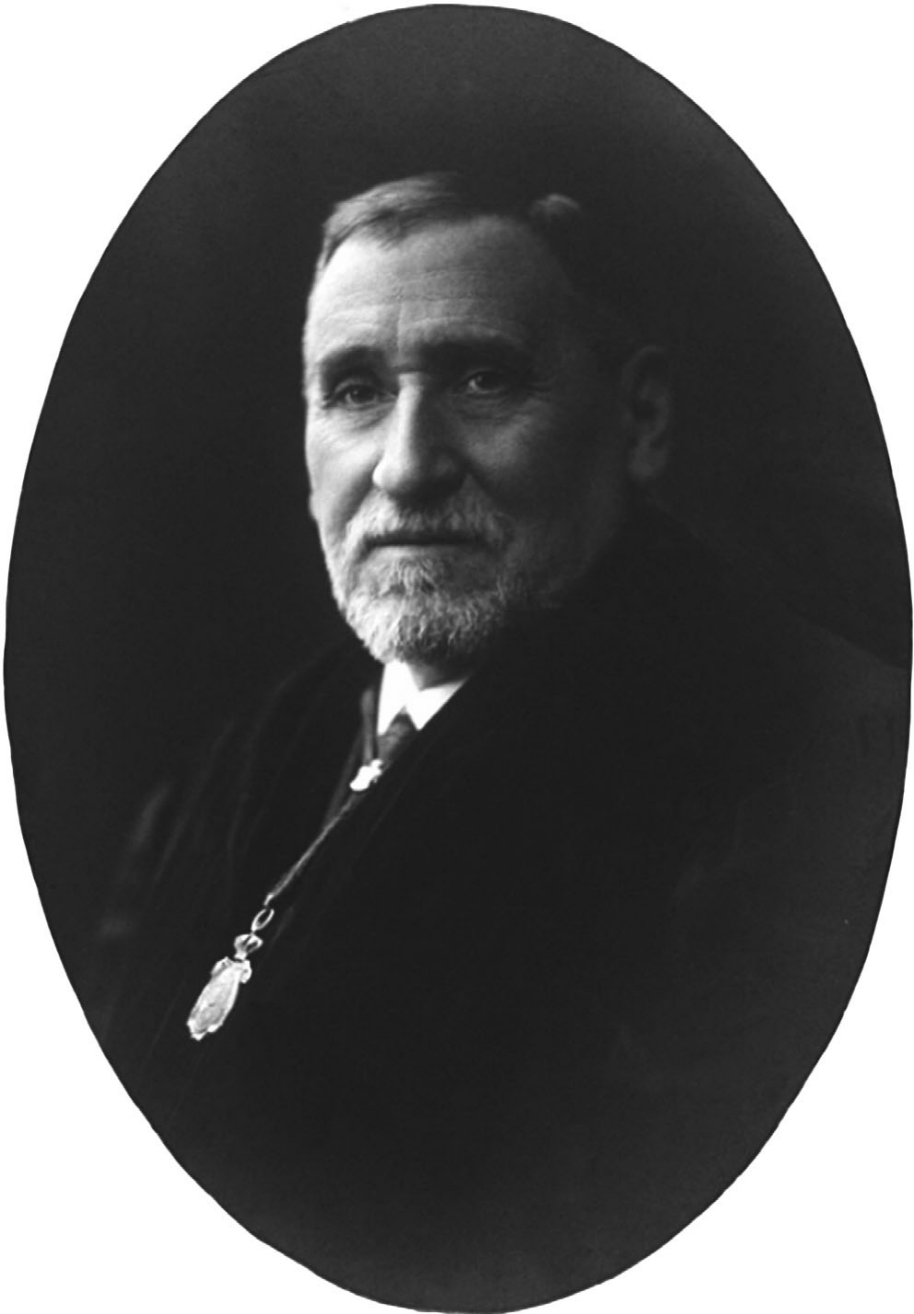
Pedro Ramón y Cajal in one of the last pictures before dying at 96 years old.

Pedro was, first and foremost, a doctor. In this sense, he differed from his brother Santiago and was more like his father (Fig. 13). During his long life, he got to experience an interesting period of medicine full of important advances, some of which he contributed to. He met Pasteur while working in the field of bacteriology, as well as the great neurologists of the period, including Waldeyer, Kölliker, Retzius, Betcherew, and his own brother, who shared his findings on the architecture of the nervous system. He saw how Lister began antisepsis, he learnt how to operate like Nelaton and ended up operating, following his techniques, but with stricter asepsis. He saw the diverse polypharmacy of the 19th century, including penicillin and other antibiotics. When he retired from his professorship in 1924, not knowing that he was yet to see much more, he wrote,

I retire from the arena proud to have lived the splendid life of our science. In my times, the most momentous advances have been made. Pathology, which was languidly oscillating between vitalism and organicism, with no clear direction, has now established a logical and indestructible base; the incognito miasmas have been embodied in germs. Rational, scientific treatment has replaced secular empiricism; the methods of pathology have created true foundations of disease.

Few doctors have practiced uninterrupted for more than half a century, but for 55 years Pedro was attending patients, until his health would no longer allow it, shortly before his death aged 96.

On 10 December 1950, Pedro Ramón y Cajal died, in his house in Zaragoza at Calle Joaquín Costa, number 12. The following day, at half past three in the afternoon there was a private burial in the family vault of the *Torrero* Cemetery, and the daily newspapers echoed the news. Here, we see an extract from the column printed in *El Noticiero de Zaragoza* (El Noticiero, 1950):

In the early hours of Sunday morning, the distinguished Doctor Pedro Ramón y Cajal died a Christian, one of the most esteemed professors to teach in our faculty of medicine, a researcher in love with scientific progress, an extraordinarily experienced doctor, an able and expert surgeon, and above all an exceptional gentleman due to his kindness, sympathy and the greatness of his soul…

## AWARDS AND ACCOLADES

- Academic numerary of the Royal Academy of Medicine of Zaragoza (1893). The following year (1984), he had to temporarily leave the Academy to move to Cádiz to take up the position of professor of histology. He rejoined in 1913 and gave his inaugural speech in 1914. In 1950, he was named honorary president.
- Martínez Molina prize for his work “*On the cerebral sensory centers in man and animals*” (1902).
- Meritorious member of the Bolonia Academy of Science (1907).
- President of the Spanish Natural History Society (1907).
- President of the Aragonese Institute of Medical Science (1907).
- President of the College of Doctors of Zaragoza (1907–1911).
- Meritorious member of the Imperial Academy of Science of Moscow (1913).
- Honorary member of the Imperial Society of Friends of Natural Sciences, Anthropology, and Ethnography of Saint Petersburg (1914).
- Corresponding member of the Royal Academy of Medicine of Madrid (1914).
- Alderman of the Esteemed Council of Zaragoza.
- Board member of the *Caja de Ahorros y Monte de Piedad* (savings bank and pawnbrokers) of Zaragoza, Aragon, and Rioja.
- Provincial medal of honor, awarded by the Esteemed Provincial Delegation of Zaragoza (1950).
- Medal of honor of the city of Zaragoza (1950).
- On 21 July 1950, the board of directors of the Zaragoza College of Doctors appointed him an honorary college member, in recognition of his “extraordinary scientific and professional merits”.
